# Cardiac hydatid cyst presenting as ventricular arrhythmia: a case report

**DOI:** 10.1186/s43044-021-00231-z

**Published:** 2021-12-07

**Authors:** Abdullah Ameen, Kiran Hilal, Asra Shaikh, Faheemullah Khan, Saulat Fatimi

**Affiliations:** 1 Department of Radiology, Aga Khan University Hospital, Stadium Road, Karachi, Pakistan; 2 Department of Cardiothoracic Surgery, Aga Khan University Hospital, Karachi, Pakistan

**Keywords:** Cardiac, Hydatid, Cyst

## Abstract

**Background:**

Hydatid disease caused by *Echinococcus granulosus* commonly involves the liver followed by lungs. Cardiac involvement is a rare occurrence and presents a challenging scenario.

**Case presentation:**

Our case describes a middle-aged gentleman who presented to the emergency room with an episode of sudden loss of consciousness resulting from ventricular tachycardia. After successful cardiac resuscitation, the patient underwent imaging that showed a lesion compatible with hydatid cyst. Surgical treatment with pharmacologic coverage was provided which resulted in good clinical outcome.

**Conclusions:**

The case highlights rare occurrence of isolated cardiac hydatid disease presenting as cardiac arrhythmia in contrast to its common routine outpatient presentation involving the liver and lungs. Good knowledge of the unusual presentations and its epidemiology is essential to the proper management of such patients.

## Background

Hydatid disease (HD) is parasitic infection caused by *Echinococcus granulosus*. It commonly involves liver (75%) and lungs (15%); however, it can involve any organ in the body [1]. Cardiac involvement by HD is rare and is reported in between 0.5% and 2% in the available literature [2]. In the heart, it commonly involves the left ventricle with differential diagnosis including cardiac tumors, congenital cysts and aneurysms [3].

Cardiac hydatid cysts have propensity to enlarge, cause pressure effect over the myocardium and result in ventricular dysfunction, mechanical obstruction of atrioventricular valves, cardiac rhythm disturbances and displacement of coronary vessels [4]. Clinical presentation varies from minor symptoms up to cardiogenic shock and sudden cardiac death [2].

**Fig. 1:**
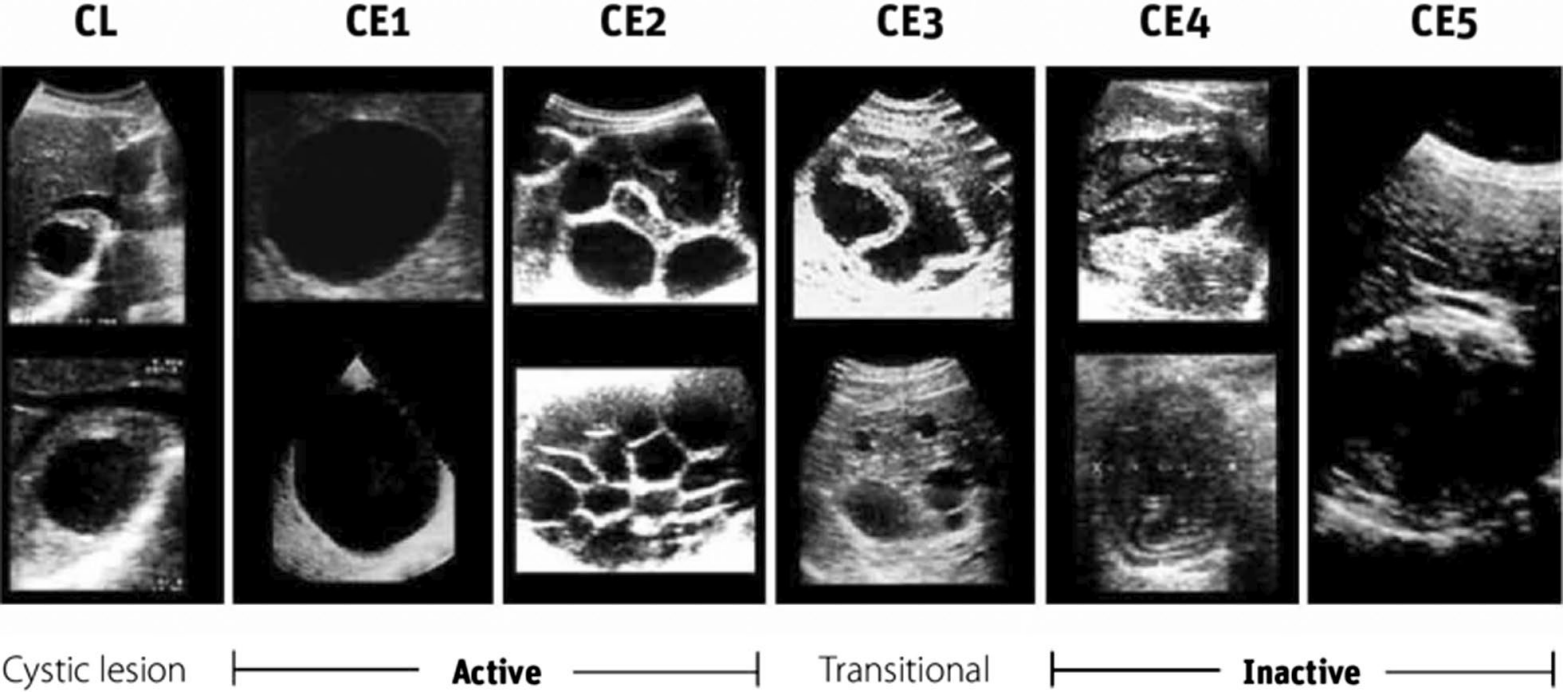
WHO-IWGE ultrasound standardized classification of echinococcal hepatic cysts © World Health Organization

Diagnosis can be made using imaging and serology. Imaging involves chest radiography which may show enlarged cardiac silhouette and mediastinal widening. Echocardiography can identify the hydatid cyst, their borders and numbers. Visceral, cardiac and mediastinal extension can be assessed using CT scan and MRI. The WHO-Informal Working Group on Echinococcosis (WHO-IWGE) developed an international ultrasound classification. According to this classification, cysts pass through CE1 (active) through CE5 (inactive) phases (Fig. 1) [5]. Standard of treatment involves surgical intervention along with pharmacotherapy. Pharmacotherapy involves administration of albendazole several days before and after surgical intervention [4].

## Case presentation

**Fig. 2:**
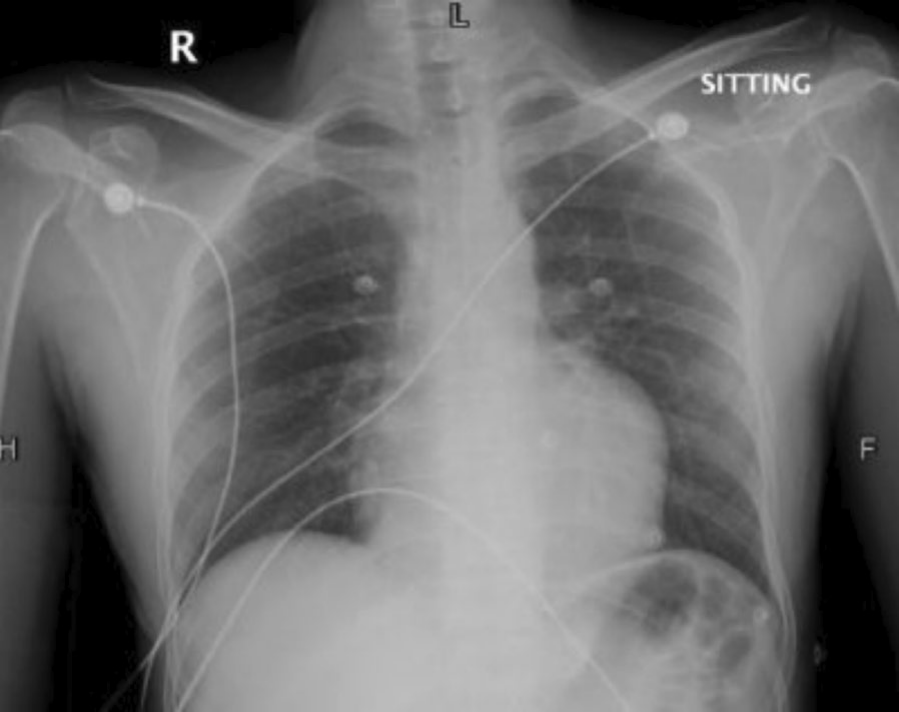
Chest X-ray shows slightly irregular and rounded left heart border

**Fig. 3:**
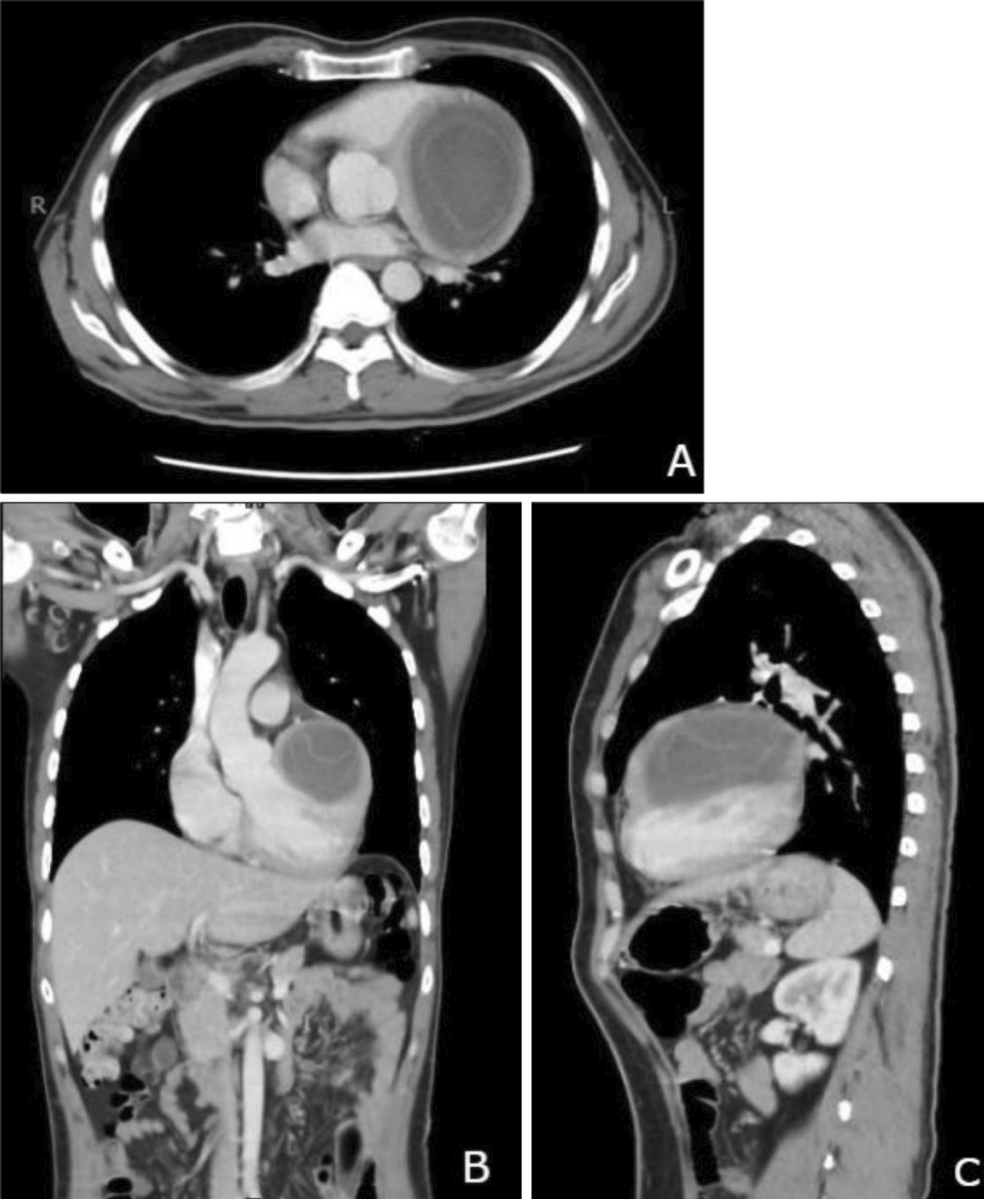
CT Chest shows a large well-defined cystic lesion with multiple internal detached membranes compressing the left ventricle.** A** through** C** (Axial, Coronal and Sagittal views)

**Fig. 4:**
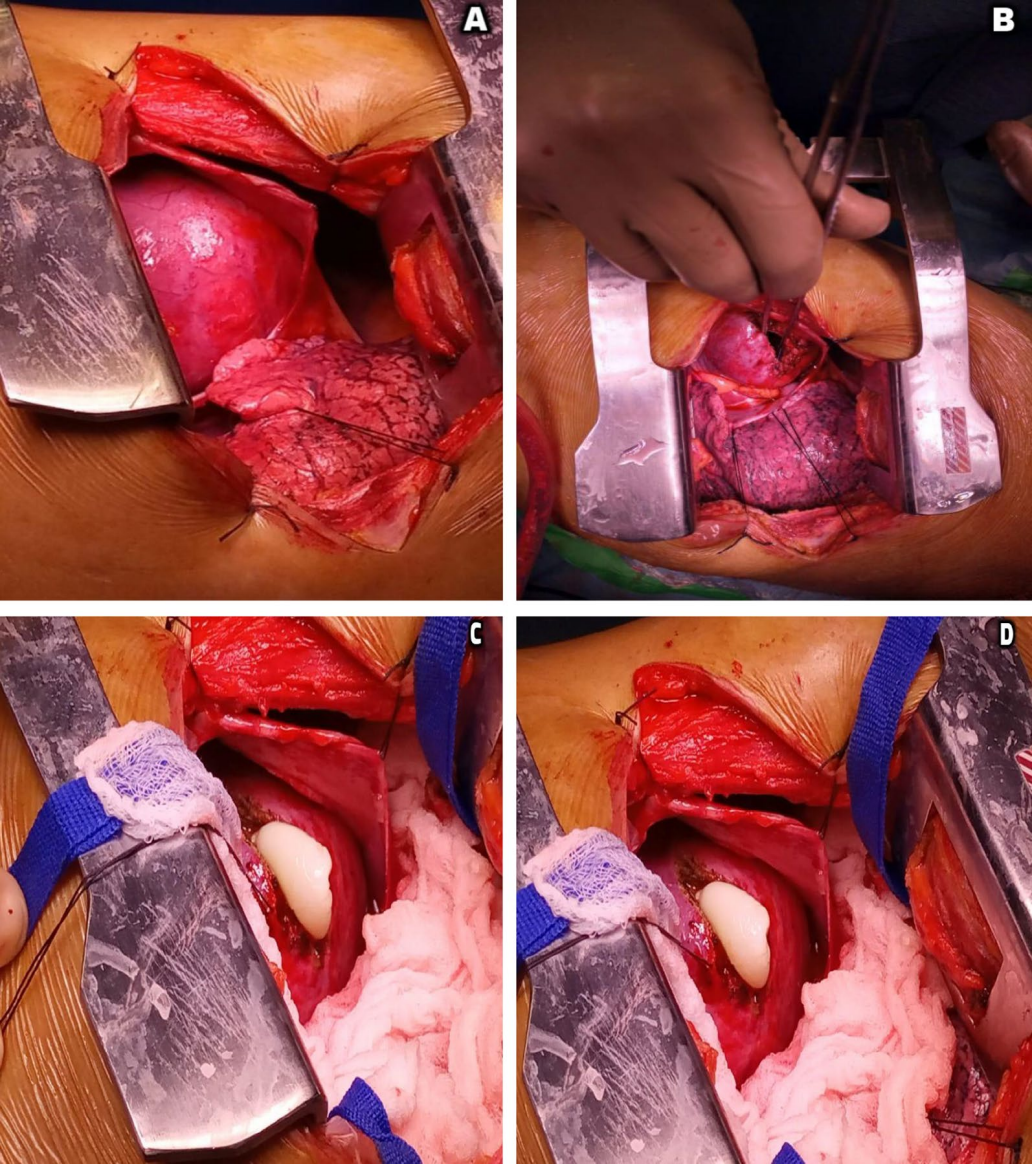
Intraoperative images of the hydatid cyst. **A** through **D**

We report a case of 35-year-old gentleman, farmer by profession, with no prior co-morbidities who developed sudden loss of consciousness. The patient presented to a local hospital with ventricular tachycardia. He was successfully resuscitated with the achievement of sinus rhythm. Cardiac catheterization showed tight distal stenosis in left circumflex artery. The patient was then referred to our tertiary care hospital where he presented in the emergency department in hemodynamically stable condition. On plain x-ray chest, the left heart border is slightly rounded (Fig. 2). Echocardiography revealed normal sized cardiac chambers with moderately reduced left ventricular systolic function and a large cystic extra cardiac mass abutting the left atrium and anterolateral wall of the left ventricle. No internal vascularity was noted in the mass. The patient then underwent CT chest examination which showed a large well-defined cystic lesion with internal detached membrane (CE3) compressing the left ventricle (Fig. 3). CT revealed hydatid cyst arising from the left ventricle. Bilateral lungs, liver and spleen were normal. ELISA-based qualitative assessment of echinococcal IgG confirmed the findings. The patient was then immediately started with albendazole and was rushed to the operating room. Left posterolateral thoracotomy was performed (Fig. 4). Pericardium was exposed, and left phrenic nerve was identified. Longitudinal incision was made on the pericardium anterior to the phrenic nerve, and pericardium was retracted. Bulging posterolateral wall of the heart was identified. Left atrium and obtuse marginal branches of left circumflex artery were identified. The hydatid cyst pseudo-capsule was delineated in the posterolateral myocardial wall, and fluid was aspirated. Then, a curvilinear incision was made on the pseudo-capsule. Large hydatid cyst with its daughter cysts was removed completely from the lateral myocardial wall. The cavity was thoroughly irrigated with hypertonic saline solution. Hemostasis was secured, and the walls of the myocardium were left open to prevent abscess formation. Pericardial and pleural chest drains were placed, and chest wall was closed in layers. Patient was extubated after 4 hours in CICU and was discharged on 4th postoperative day. He was kept on albendazole therapy for a year. Patient was seen at regular intervals in the clinic. He remained well with no recurrence of disease. Ethical approval for the publication of this case was granted by the Ethics Review Committee vides “2021-5869-15470.” Informed written consent for participation was obtained from the patient.

## Discussion

Left ventricle is the most common site of the cardiac hydatid cyst (60%) [6], as was seen in our patient. This frequent involvement of left ventricle by the hydatid cyst is secondary to increased muscle mass and perfusion [7]. Left ventricular involvement by hydatid cyst can mimic left ventricular aneurysm [8]. There is variation in clinical presentation of the patients according to the number, size and site which also makes an early diagnosis difficult. In the early phases, the growth of cyst is in between the cardiac fibers not resulting in any significant signs or symptoms; however, later it may result in pericardial pain, dyspnea, invasion of the surrounding structures or obstruction of the flow and invasion of cardiac conduction system resulting in cardiac arrhythmias or heart block [8, 9]. This highlights the fact that early resuscitation, prompt imaging and cystectomy are the utmost important things while dealing with such cases. High index of suspicion and prompt imaging were the key to reach diagnosis in our case (the endemic nature of HD in this part of the world).

Most of the time a normal cardiothoracic ratio is seen on chest radiographs [10]. We saw that in our case. There is variation in electrocardiographic findings according to the site of cyst. Echocardiography is an easy and useful technique in diagnosis of cardiac hydatid cysts; however, CT and MRI are essential for evaluation of the precise location, extent and anatomic relationships of the cyst [6, 11]. Serology is not considered reliable with false-positive and false-negative results approaching 30% for Casoni test. ELISA, on the other hand, has reported sensitivity of 91% and specificity of 82% [12, 13]. A positive ELISA test for echinococcal antibodies confirms the diagnosis, as was seen in our patient.

Treatment consists of surgical excision and pharmacologic treatment. Anthelminthic drugs (e.g., albendazole) have been used in the preoperative and postoperative periods since long [14]. Scolicidal solutions including iodine, ethanol, methylene blue or hypertonic saline are used to reduce risk of leakage of cystic fluid [15, 16]. Post-surgery, the duration of antihelminthic therapy is variable. In our patient, albendazole was prescribed to the patient postoperatively with good clinical outcome.

## Conclusions

Isolated cardiac involvement by the HD is a rare finding, as noted in our case. Increased awareness of this entity is essential for physicians and diagnosticians, especially in endemic areas. Routine screening echocardiography is the key followed by cross-sectional imaging which has a high diagnostic value in order to avoid the potential life threatening complication of disease. Surgery in these cases requires good surgical hands, especially when the presentation is through the emergency room.

## Data Availability

Data sharing is not applicable to this article as no datasets were generated or analyzed during the current study.

## Abbreviations

HD: Hydatid disease
CT: Computed tomography
MRI: Magnetic resonance imaging
ELISA: Enzyme-linked immunosorbent assay
CICU: Coronary intensive care unit
IV: Intravenous
